# Supplementary material for: Assessment of Food Safety Knowledge and Behaviors of Cancer Patients Receiving Treatment
Source: Nutrients. 2019 Aug 14;11(8):1897. doi: 10.3390/nu11081897 (PMC6722877; doi:10.3390/nu11081897)
Supplement: Supplementary file 1 [file nutrients-11-01897-s001.zip › Assessment of Food Safety Knowledge and Behaviors of Cancer Patients Receiving Treatment Supplemental Material 2. Survey.docx]

**We appreciate your willingness to complete this survey. Please start from page 2. This page is for *official* use *only*.**

| **Forms and Procedures** | **Check**  **Initials** |
| --- | --- |
| Demographic Questionnaire |  |
| Disease Characteristics |  |
| Medication and Treatment Adherence |  |
| Food Security Questionnaire |  |
| Quality of Life |  |
| Risk Perception |  |
| Food Preferences |  |
| Food Safety |  |
| Pay Participant |  |
| Progress Notes (Below) |  |
|  |  |
|  |  |
|  |  |

**A. DEMOGRAPHIC QUESTIONNAIRE**

**For these questions, please mark your answers** (**⌧**or **🗹**) **or write your answers in the spaces provided.**

1. Gender: ⬜_1_ Male ⬜_2_ Female

2. Age: ⬜_1_ 18-29 ⬜_2_ 30-39 ⬜_3_ 40-49 ⬜_4_ 50-59 ⬜_5_ 60-69 ⬜_6_ ≥ 70

3. Which of the following best describes your ethnic background?

⬜_1_ Asian ⬜_2_ Black/African ⬜_3_ Hispanic

⬜_4_ Native American ⬜_5_ White Non-Hispanic ⬜_6_ Other (specify___________)

4. Whish of the following best describes your marital Status?

⬜_1_ Married ⬜_2_ Single/Never Married

⬜_3_ Divorced/Widowed ⬜_4_ Other ________

5. What is your highest level of education? ⬜_1_ < High school ⬜_2_ High school or GED

⬜_3_ 1-2 years college ⬜_4_ ≥College degree

6. What is your employment Status? ⬜_1_ 40+ hours a week ⬜_2_ ≤40 hours a week

⬜_3_ Homemaker ⬜_4_ Retired ⬜_5_ Unemployed

7. What is your household’s monthly income? ⬜_1_ <$1000 ⬜_2_ $1000-$1999

⬜_3_ $2000-$2999 ⬜_4_ $3000-$3999 ⬜_5_ ≥$4000

8. How many people live in your household? ⬜_1_ One ⬜_2_ Two ⬜_3_ Three

⬜_4_ Four ⬜_5_ ≥Five

9. How many children (<18yrs) live in household? ⬜_1_ One ⬜_2_ Two ⬜_3_ Three

⬜_4 ≥_Four ⬜_5_ None

10. What is your health insurance status? ⬜_1_ None ⬜_2_ Private

⬜_3_ Public non-Medicare ⬜_4_ Medicare

11. Have you had to borrow money to pay for healthcare? ⬜_1_ Yes ⬜_2_ No

12. Have you had to pay your bills late due to medical expenses? ⬜_1_ Yes ⬜_2_ No

13. Which food assistance program do you participate in? ⬜_1_ SNAP/Food Stamps ⬜_2_ WIC ⬜_3_ Other __________ ⬜_4_ None

14. Receive foods from a food bank, a food pantry, or a soup kitchen: ⬜_1_ Yes ⬜_2_ No

14a. If yes, please specify________________________________

15. What is the best description for where you live? ⬜_1_ Urban ⬜_2_ Rural ⬜_3_ Suburban

16. What is your smoking status? ⬜_1_ Current smoker ⬜_2_ Former smoker

⬜_3_ Never smoked

16a. If current smoker, how often do you smoke? ⬜_1_ Daily ⬜_2_ 4-6 days/week

⬜_3_ 2-3 days/week ⬜_4_ 1 day/week

16b. How many cigarettes per day do you smoke? _________________

17. Do you use any recreational drugs? ⬜_1_ Yes ⬜_2_ No

17a.If yes please specify:­­­­­­­­­­­­­________________________________________

18. Do you drink alcohol? ⬜_1_ Yes ⬜_2_ No

18a. If yes how often? ⬜_1_ Daily ⬜_2_ 4-6 days/week

⬜_3_ 2-3 days/week ⬜_4_ 1 day/week

18b. On the days you drank alcohol, how many drinks do you usually have?

------ Beer drinks

------ Wine drinks

------ Liquor drinks

------Total drinks

**B. DISEASE CHARACTERISTICS**

1. Type of cancer diagnose with: ______________________________

1a Time since disease diagnosis: ⬜_1_ 0-6 months ⬜_2_ 7 months – less than 2 yrs

⬜_3_ 2 yrs- less than 5 yrs ⬜_4_ 5 yrs or more

1b. Stage of disease: ⬜_1_ I ⬜_2_ II ⬜_3_ III ⬜_4_ IV ⬜_5_ Unknown

2. Type of cancer treatment received? ⬜_1_ Chemotherapy ⬜_2_ Radiation therapy ⬜_3_ Hormone therapy ⬜_4_ Surgery

⬜_5_ Combinationtherapy ⬜_6_ Other___________

**C. MEDICATION AND TREATMENT ADHERENCE**

1. Do you receive oral medications as part of your cancer treatment? ⬜_1_Yes ⬜_2_ No

***If no Skip to Question 8***

**Most people diagnosed with cancer have many pills to take at different times during the day. Many people find it hard to always remember their pills. We need to understand how people with cancer are really doing with their pills. Please tell us what you are actually doing. Don’t worry about telling us that you don’t take all your pills. We need to know what is really happening, not what you think we “want to hear”**

2. During the past 4 days, on how many days have you missed taking all your doses?

⬜_1_ None ⬜_2_ One day ⬜_3_ Two days ⬜_4_ Three days ⬜_5_ Four days

3. Most cancer medications need to be taken on a schedule, such as “2 times a day” or “3 times a day” or “every 8 hours.” How closely did you follow your specific schedule over the last four days?

⬜_1_Never ⬜_2_ Some of the time ⬜_3_ About half of the time

⬜_4_Most of the time ⬜_5_ All of the time

4. Do any of your cancer medications have special instructions, such as “take with food” or “on an empty stomach” or “with plenty of fluids?”

⬜_1_ Yes ⬜_2_ No

If Yes, how often did you follow those special instructions over the last four days?

⬜_1_Never ⬜_2_ Some of the time ⬜_3_ About half of the time

⬜_4_Most of the time ⬜_5_ All of the time

5. Some people find that they forget to take their pills on the weekends. Did you miss any of your cancer medications last weekend— last Saturday or Sunday?

⬜_1_ Yes ⬜_2_ No

6. When was the last time you missed any of your medications? Check one.

⬜_1_ Within the past week ⬜_2_ 1-2 weeks ago ⬜_3_ 2-4 weeks ago

⬜_4_ 1-3 months ago ⬜_5_ More than 3 months ago

⬜_6_ Never skip medications or not applicable

7**. People may miss taking their medications for various reasons. Here is a list of possible reasons why you may miss taking your medications**. **How often have you missed taking your medications because you: (Circle one response for each question.)**

|  | **Never** | **Rarely** | **Sometimes** | **Often** |
| --- | --- | --- | --- | --- |
| a. Were away from home? | 1 | 2 | 3 | 4 |
| b. Were busy with other things? | 1 | 2 | 3 | 4 |
| c. Simply forgot? | 1 | 2 | 3 | 4 |
| d. Had too many pills to take? | 1 | 2 | 3 | 4 |
| e. Wanted to avoid side effects? | 1 | 2 | 3 | 4 |
| f. Didn’t want others to notice you taking meds? | 1 | 2 | 3 | 4 |
| g. Had a change in daily routine? | 1 | 2 | 3 | 4 |
| h. . Felt like the drug was toxic/harmful? | 1 | 2 | 3 | 4 |
| i. Fell asleep/slept through dose time? | 1 | 2 | 3 | 4 |
| j. Felt sick or ill? | 1 | 2 | 3 | 4 |
| k. Felt depressed/overwhelmed? | 1 | 2 | 3 | 4 |
| l. Had problems taking pills at specified times (with meals,on empty stomach, etc.)? | 1 | 2 | 3 | 4 |
| m. Ran out of pills? | 1 | 2 | 3 | 4 |
| n. Felt good? | 1 | 2 | 3 | 4 |

8. Have you ever missed your treatment appointments? ⬜_1_Yes ⬜_2_ No

9a. If yes, how many times within the past treatment cycle? ⬜_1_ 0-1 ⬜_2_ 2-4 ⬜_3_ >5

**D. US ADULT FOODSECURITY SURVEY MODULE**

**Below are several statements that people have made about their food situation. For these statements, please indicate whether the statement was often true, sometimes true, or never true for (you/your household) in the last 12 months**

1. The first statement is “(I/We) worried whether (my/our) food would run out before (I/we) got money to buy more.” Was that often true, sometimes true, or never true for (you/your household) in the last 12 months?

[ ] Often true [ ] Sometimes true [ ] Never true [ ] DK or Refused

2.“The food that (I/we) bought just didn’t last, and (I/we) didn’t have money to get more.” Was that often, sometimes, or never true for (you/your household) in the last 12 months?

[ ] Often true [ ] Sometimes true [ ] Never true [ ] DK or Refused

3.“(I/we) couldn’t afford to eat balanced meals.” Was that often, sometimes, or never true for (you/your household) in the last 12 months?

[ ] Often true [ ] Sometimes true [ ] Never true [ ] DK or Refused

***If affirmative response (i.e., "often true" or "sometimes true") to one or more of Questions 1-3, then continue to Adult Stage 2; otherwise skip to SECTION E of survey.***

*Adult Stage 2: Questions*

AD1. In the last 12 months, since last (name of current month), did (you/you or other adults in your household) ever cut the size of your meals or skip meals because there wasn't enough money for food?

[ ] Yes [ ] No (Skip AD1a) [ ] DK (Skip AD1a)

AD1a. If yes, how often did this happen [ ] Almost every month

[ ] Some months but not every month [ ] Only 1 or 2 months [ ] DK

AD2. In the last 12 months, did you ever eat less than you felt you should because there wasn't enough money for food?

[ ] Yes [ ] No [ ] DK

AD3. In the last 12 months, were you every hungry but didn't eat because there wasn't enough money for food?

[ ] Yes [ ] No [ ] DK

AD4. In the last 12 months, did you lose weight because there wasn't enough money for food?

[ ] Yes [ ] No [ ] DK

***If affirmative response to one or more of questions AD1-AD4, continue to Adult Stage 3.***

Adult Stage 3:

AD5. In the last 12 months, did (you/you or other adults in your household) ever not eat for a whole day because there wasn't enough money for food?

[ ] Yes [ ] No (Skip AD5a) [ ] DK (Skip AD5a)

**AD5a. If yes, how often did this happen**?

[ ] Almost every month [ ] Some months but not every month

[ ] Only 1 or 2 months [ ] DK

**E. QUALITY OF LIFE (EORTC QLQ - BR23)**

**Patients sometimes report that they have the following symptoms or problems. Please indicate the extent to which you have experienced these symptoms or problems during the past week.**

| **During the past week: Not at A Quite Very** |
| --- |
| **All Little a Bit Much** |
| 1. Did you have a dry mouth? 1 2 3 4 |
| 2. Did food and drink taste different than usual? 1 2 3 4 |
| 3. Were your eyes painful, irritated or watery? 1 2 3 4 |
| 4. Have you lost any hair? 1 2 3 4 |
| 5. Answer this question only if you had any hair loss:  Were you upset by the loss of your hair? 1 2 3 4 |
| 6. Did you feel ill or unwell? 1 2 3 4 |
| 7. Did you have hot flushes? 1 2 3 4 |
| 8. Did you have headaches? 1 2 3 4 |
| 9. Have you felt physically less attractive  as a result of your disease or treatment? 1 2 3 4 |
| 10. Have you been feeling less feminine as a   result of your disease or treatment? **(if male, skip to 11)** 1 2 3 4 |
| 11. Did you find it difficult to look at yourself naked? 1 2 3 4 |
| 12. Have you been dissatisfied with your body? 1 2 3 4 |
| 13. Were you worried about your health in the future? 1 2 3 4 |
| **During the past four weeks: Not at A Quite Very** |
| **All Little a Bit Much** |
| 14. To what extent were you interested in sex? 1 2 3 4 |
| 15. To what extent were you sexually active? 1 2 3 4  (with or without intercourse) |
| 16. Answer this question only if you have been sexually 1 2 3 4  active: To what extent was sex enjoyable for you? |
| **During the past week: Not at A Quite Very** |
| **All Little a Bit Much** |
| 17. Did you have any pain in your arm or shoulder? 1 2 3 4 |
| 18. Did you have a swollen arm or hand? 1 2 3 4 |
| 19. Was it difficult to raise your arm or to move  it sideways? 1 2 3 4 |
| **If diagnosed with breast cancer, complete questions 20-23. If not, skip to section F.** |
| 20. Have you had any pain in the area of your affected breast? 1 2 3 4 |
| 21. Was the area of your affected breast swollen? 1 2 3 4 |
| 22. Was the area of your affected breast oversensitive? 1 2 3 4 |
| 23. Have you had skin problems on or in the area of |
| your affected breast (e.g., itchy, dry, flaky)? 1 2 3 4 |

**F. RISK PERCEPTION, ATTITUDES AND BEHAVIORS**

| Items | Strongly disagree | Disagree | Neutral | Agree | Strongly agree |
| --- | --- | --- | --- | --- | --- |
| I think contamination of food by bacteria or viruses is a serious problem | 1 - - - - 2 - - - - 3 - - - - 4 - - - - 5 | | | | |
| I am knowledgeable about how to keep the food I prepare and eat at home safe | 1 - - - - 2 - - - - 3 - - - - 4 - - - - 5 | | | | |
| I am knowledgeable about safe food choices when I eat outside the home | 1 - - - - 2 - - - - 3 - - - - 4 - - - - 5 | | | | |
| Because I am cancer survivor I am at an increased risk of getting foodborne illness or food poisoning | 1 - - - - 2 - - - - 3 - - - - 4 - - - - 5 | | | | |

**This is a survey about the ways you fix food. It is not a test, and there are no wrong answers. When answering questions, please circle the number that applies to the way you usually do things.**

| Items | Never | Rarely | Some of the time | Most of the time | Always | |
| --- | --- | --- | --- | --- | --- | --- |
|  |  | | | | |  |
| I am not concerned if I thaw perishable foods on the kitchen counter | 1 - **-** - - 2 - **-** - - 3 - **-** - - 4 - **-** - - 5 | | | | |  |
| Cooking and eating eggs that have firm yolks and whites is important to me for safety | 1 - **-** - - 2 - **-** - - 3 - **-** - - 4 - **-** - - 5 | | | | |  |
| Drinking pasteurized apple juice or cider is important to me for safety | 1 - **-** - - 2 - **-** - - 3 - **-** - - 4 - **-** - - 5 | | | | |  |
| After cutting raw meat or chicken, I like to wash the cutting board, knife, and counter top with hot soapy water before continuing cooking | 1 - **-** - - 2 - **-** - - 3 - **-** - - 4 - **-** - - 5 | | | | |  |
| I am not interested in using a meat thermometer | 1 - **-** - - 2 - **-** - - 3 - **-** - - 4 - **-** - - 5 | | | | |  |
| I don’t worry that I may get sick if I eat alfalfa and other raw sprouts | 1 - **-** - - 2 - **-** - - 3 - **-** - - 4 - **-** - - 5 | | | | |  |
| I am worried that I may get sick if I eat hot dogs right out of the package | 1 - **-** - - 2 - **-** - - 3 - **-** - - 4 - **-** - - 5 | | | | |  |
| Using cheese and yogurt made only from pasteurized milk is important to me | 1 - **-** - - 2 - **-** - - 3 - **-** - - 4 - **-** - - 5 | | | | |  |
| I am concerned that I may get sick if I eat raw oysters | 1 - **-** - - 2 - **-** - - 3 - **-** - - 4 - **-** - - 5 | | | | |  |
| I don’t worry about keeping the refrigerator at or below 40 degrees Fahrenheit | 1 - **-** - - 2 - **-** - - 3 - **-** - - 4 - **-** - - 5 | | | | |  |
| I don’t worry about washing my hands after playing with my pets | 1 - **-** - - 2 - **-** - - 3 - **-** - - 4 - **-** - - 5 | | | | |  |
| It is not important to cover a cut or sore on my hand before I prepare food | 1 - **-** - - 2 - **-** - - 3 - **-** - - 4 - **-** - - 5 | | | | |  |
| Refrigerating food such as rice and beans overnight before serving them the following day is not important to me | 1 - **-** - - 2 - **-** - - 3 - **-** - - 4 - **-** - - 5 | | | | |  |
| There is no need to store eggs in a refrigerator, room temperature is just fine | 1 - **-** - - 2 - **-** - - 3 - **-** - - 4 - **-** - - 5 | | | | |  |

| Items | Never | Rarely | Some of the time | Most of the time | Always |  |
| --- | --- | --- | --- | --- | --- | --- |
| I wash my hands with soap and warm running water before preparing food | 1 - - - - 2 - - - - 3 - - - - 4 - - - - 5 | | | | | |
| After playing with a pet and before getting a snack, I wash my hands with soap and warm running water. | 1 - - - - 2 - - - - 3 - - - - 4 - - - - 5 | | | | | |
| After cutting raw meat, chicken, or seafood, I wash all items that came in contact with the raw food (e.g., cutting board, knife, counter top) with hot, soapy water before I continue cooking. | 1 - - - - 2 - - - - 3 - - - - 4 - - - - 5 | | | | | |
| I thoroughly rinse fresh vegetables under running water before eating them | 1 - - - - 2 - - - - 3 - - - - 4 - - - - 5 | | | | | |
| I wash the plate used to hold raw meat, poultry, or seafood with hot, soapy water before returning cooked food to the plate OR I use a clean plate. | 1 - - - - 2 - - - - 3 - - - - 4 - - - - 5 | | | | | |
| I wash my hands with soap and warm running water after working with raw meat, chicken, or seafood and before I continue cooking | 1 - - - - 2 - - - - 3 - - - - 4 - - - - 5 | | | | | |
| I clean countertops with hot soapy water after preparing food | 1 - - - - 2 - - - - 3 - - - - 4 - - - - 5 | | | | | |
| I leave cooked foods, such as rice or beans, on the stovetop overnight to be used the next day | 1 - - - - 2 - - - - 3 - - - - 4 - - - - 5 | | | | | |
| I put frozen meat and poultry on the counter in the morning so it will be thawed and ready to cook in the evening | 1 - - - - 2 - - - - 3 - - - - 4 - - - - 5 | | | | | |
| I store my eggs at room temperature | 1 - - - - 2 - - - - 3 - - - - 4 - - - - 5 | | | | | |
| When I cook fish, I check that the flesh flakes easily with a fork before serving | 1 - - - - 2 - - - - 3 - - - - 4 - - - - 5 | | | | | |
| I use a thermometer to check the temperature of my fridge | 1 - - - - 2 - - - - 3 - - - - 4 - - - - 5 | | | | | |
| I prepare food for others when I have diarrhea | 1 - - - - 2 - - - - 3 - - - - 4 - - - - 5 | | | | | |
| I use a thermometer to determine if leftovers have been reheated enough | 1 - - - - 2 - - - - 3 - - - - 4 - - - - 5 | | | | | |
| I use a thermometer to determine if chicken breasts have been cooked enough | 1 - - - - 2 - - - - 3 - - - - 4 - - - - 5 | | | | | |

**G. FOOD PREFERENCES**

**Please circle YES or NO.**

| **Do you eat the following foods?** |  |  |
| --- | --- | --- |
| Rare hamburger | YES | NO |
| Eggs with runny yolks | YES | NO |
| Raw oysters/oysters on the half shell | YES | NO |
| Raw fish | YES | NO |
| Homemade cookie dough | YES | NO |
| Alfalfa or other raw sprouts | YES | NO |
| Ceviche (marinated raw fish) | YES | NO |
| Sushi (made with raw fish) | YES | NO |
| Restaurant salad bars | YES | NO |
| Cold hot dogs | YES | NO |
| Soft cheese like Brie, Camembert, and queso fresco | YES | NO |
| Smoked fish served cold without reheating | YES | NO |
| Cold deli meats | YES | NO |

**H. FOOD SAFETY KNOWLEDGE**

**Please indicate whether you agree/disagree with the following statements.**

| **General Food Safety** |  |
| --- | --- |
| All harmful bacteria are destroyed by thorough and complete cooking. | Disagree/ agree |
| Moldy hard cheddar cheese is safe to eat if you scratch the mold off the surface of the cheese. | Disagree/ agree |
| Organically grown produce is less likely to cause foodborne illness than conventionally grown produce. | Disagree/ agree |
| Pesticide residues are the most serious food safety problem. | Disagree/ agree |
| Young children are more vulnerable to foodborne illnesses than teenagers or adolescents. | Disagree/ agree |
| Unsafe foods can be identified by the way they look and smell. | Disagree/ agree |
| Food allergies are a serious food safety problem. | Disagree/ agree |
| Food home illness outbreaks are associated with eating all types of food. | Disagree/ agree |
| Bacteria and viruses found in food can make you sick. | Disagree/ agree |
| Disease-causing bacteria can be found on food. | Disagree/ agree |
| It can take only a small number of harmful bacteria to make a person sick. | Disagree/ agree |
| **Food handling** |  |
| After cutting up raw meat or chicken, you should wipe off cutting board with wet dishcloth or sponge before using the board to cut produce. | Disagree/ agree |
| It is safe to store fresh produce below raw meat and poultry in the fridge. | Disagree/ agree |
| It is important to wash hands after "cracking" an egg. | Disagree/ agree |
| When grocery shopping, raw meat, fish and/or poultry should be packed separately from ready-to-eat foods from the deli or produce area. | Disagree/ agree |
| In the kitchen, food can become contaminated with harmful bacteria during handling and storage. | Disagree/ agree |
| The sauce that was used to marinate raw chicken can be refrigerated and used again safely. | Disagree/ agree |
| Since a food thermometer has a metal stem, it does not need to be sanitized after using it. | Disagree/ agree |
| It is safe to place cooked meat on the same unwashed plate you used for the uncooked meat. | Disagree/ agree |
| **Food handling** |  |
| To be safe to eat, the temperature of stuffing cooked inside a turkey should be at least 145° F. | Disagree/ agree |
| Chicken breasts should be cooked until the temperature in the middle is 180° F. (Note recent change to 165° F) | Disagree/ agree |
| Cooked rice held at room temperature for more than 4 hours is safe to eat. | Disagree/ agree |
| I can always tell that my hamburger is completely cooked by its color. | Disagree/ agree |
| It is safe to use unpasteurized eggs in recipes that will not be cooked. | Disagree/ agree |
| Cooked meat held at room temperature for more than 2 hours is safe to eat. | Disagree/ agree |
| Using a food thermometer is the best way of knowing that food is thoroughly cooked. | Disagree/ agree |
| It is safe to eat raw cookie dough or cake batter that contains raw eggs. | Disagree/ agree |
| **Food storage (chill)** |  |
| One of the most common causes of foodborne illness is failure to properly cool food. | Disagree/ agree |
| Deli meats or cold cuts sliced at the deli counter are safe to eat for seven days after purchase. | Disagree/ agree |
| It is safe to leave hot, thoroughly cooked food on the counter to completely cool to room temperature before putting it in the refrigerator. | Disagree/ agree |
| Meat that has been handled and/or prepared properly can be kept in the freezer for 6 months and still be safe to eat. | Disagree/ agree |
| The temperature of a home refrigerator should be at 40^o^ F or below. | Disagree/ agree |
| Disease-causing bacteria can survive and/or grow at refrigerator temperatures. | Disagree/ agree |
| It is safe to leave meat on the counter to thaw. | Disagree/ agree |
| If a green bean casserole is left on the kitchen counter overnight, it is safe to eat if it is properly reheated. | Disagree/ agree |
| **Clean up (Cleaning/Hygiene)** |  |
| Countertops may be sanitized by washing with soap and water. | Disagree/ agree |
| Hand sanitizers are the best way to wash your hands. | Disagree/ agree |
| You should wash the outside of a cantaloupe before cutting it. | Disagree/ agree |
| You should wash your hands with warm, soapy water for at least 15 seconds before starting to prepare food. | Disagree/ agree |
| It is safe to eat a snack while you are preparing food. | Disagree/ agree |
| If you use a dishcloth to wipe up liquid from raw meat or chicken, it is safe to use the cloth for washing dishes if you rinse the cloth in hot water. | Disagree/ agree |
| Sponges will not be contaminated with bacteria since they are used to wash utensils with soap and water. | Disagree/ agree |
| It is safe to use the same spoon to taste and then stir the food without washing the spoon. | Disagree/ agree |
| It is safe to use a cloth towel to clean up spills on kitchen surfaces and then use it to dry off washed fresh fruits or vegetables. | Disagree/ agree |
| After handling raw meat, fish and/or poultry, wiping hands on a paper towel is sufficient to clean hands. | Disagree/ agree |

**This is a survey about the ways you acquire and consume food. It is not a test, and there are no wrong answers. When answering questions, please circle the number that applies.**

| Items | Never | | Rarely | Some of the time | Most of the time | Always |
| --- | --- | --- | --- | --- | --- | --- |
| I cook with other people | | 1 - - - - 2 - - - - 3 - - - - 4 - - - - 5 | | | | |
| I borrow food from other people | | 1 - - - - 2 - - - - 3 - - - - 4 - - - - 5 | | | | |
| I get food from workplace | | 1 - - - - 2 - - - - 3 - - - - 4 - - - - 5 | | | | |
| I acquire discarded food | | 1 - - - - 2 - - - - 3 - - - - 4 - - - - 5 | | | | |
| I acquire food from private individuals | | 1 - - - - 2 - - - - 3 - - - - 4 - - - - 5 | | | | |
| I seek roadkill | | 1 - - - - 2 - - - - 3 - - - - 4 - - - - 5 | | | | |
| I hunt or fish | | 1 - - - - 2 - - - - 3 - - - - 4 - - - - 5 | | | | |
| I purchase food from private individuals | | 1 - - - - 2 - - - - 3 - - - - 4 - - - - 5 | | | | |
| I purchase expired foods | | 1 - - - - 2 - - - - 3 - - - - 4 - - - - 5 | | | | |
| I purchase nearly expired foods | | 1 - - - - 2 - - - - 3 - - - - 4 - - - - 5 | | | | |
| I purchase foods in dented or damaged packages | | 1 - - - - 2 - - - - 3 - - - - 4 - - - - 5 | | | | |
| I remove slime from lunch meat | | 1 - - - - 2 - - - - 3 - - - - 4 - - - - 5 | | | | |
| I remove mold from cheese | | 1 - - - - 2 - - - - 3 - - - - 4 - - - - 5 | | | | |
| I remove mold from grains | | 1 - - - - 2 - - - - 3 - - - - 4 - - - - 5 | | | | |
| I remove insects from grains | | 1 - - - - 2 - - - - 3 - - - - 4 - - - - 5 | | | | |
| I remove spoiled parts of fruits/vegetables | | 1 - - - - 2 - - - - 3 - - - - 4 - - - - 5 | | | | |
| I store perishables inadequately | | 1 - - - - 2 - - - - 3 - - - - 4 - - - - 5 | | | | |
| I eat spoiled food | | 1 - - - - 2 - - - - 3 - - - - 4 - - - - 5 | | | | |
| I eat expired food | | 1 - - - - 2 - - - - 3 - - - - 4 - - - - 5 | | | | |
| I eat non-food items | | 1 - - - - 2 - - - - 3 - - - - 4 - - - - 5 | | | | |
| I eat other people’s leftovers | | 1 - - - - 2 - - - - 3 - - - - 4 - - - - 5 | | | | |
| I eat roadkill | | 1 - - - - 2 - - - - 3 - - - - 4 - - - - 5 | | | | |
| I eat pet food | | 1 - - - - 2 - - - - 3 - - - - 4 - - - - 5 | | | | |
