# Supplementary material for: Assessment of Food Safety Knowledge and Behaviors of Cancer Patients Receiving Treatment
Source: Nutrients. 2019 Aug 14;11(8):1897. doi: 10.3390/nu11081897 (PMC6722877; doi:10.3390/nu11081897)
Supplement: Supplementary file 1 [file nutrients-11-01897-s001.zip › Assessment of Food Safety Knowledge and Behaviors of Cancer Patients Receiving Treatment Supplemental Materials 1. Patient Response Frequencies.docx]

| **Demographic characteristics** | **Categories** | **n** | **%** |
| --- | --- | --- | --- |
|  |  |  |  |
| *Gender* | Female | 192 | 66.9 |
|  | Male | 95 | 33.1 |
| *Age* | 18-29 | 7 | 2.4 |
|  | 30-39 | 19 | 6.6 |
|  | 40-49 | 38 | 13.3 |
|  | 50-59 | 97 | 33.9 |
|  | 60-69 | 84 | 29.4 |
|  | >70 | 41 | 14.3 |
| *Race* | Asian | 2 | 0.7 |
|  | Black/ African  Hispanic  Native American  White  Other | 27  2  3  250  3 | 9.4  0.7  1.0  87.1  1.0 |
| *Marital Status* | Married  Single  Divorced/Widowed  Other | 175  31  77  5 | 60.8  10.8  26.7  1.7 |
| *What is your highest level of education?*  *What is your employment status?*  *What is your household’s monthly income?*  *How many people live in your household?*  *How many children (<18yrs) live in your household?*  *What is your health insurance status?*  *Have you had to borrow money to pay for healthcare?*  *Have you had to pay your bills late due to medical expenses?*  *Which food assistance programs do you participate in?*  *Receive foods from a food bank, a food pantry, or a soup kitchen*  *What is the best description for where you live?*  *What is your smoking status?*  *If current smoker, how often do you smoke?*  *Do you use any recreational drugs?*  *Do you drink alcohol?*  *If yes, how often?* | <H.S.  H.S./GED  1-2 college  >or= college  40+  <or=40  Home  Retired  None  <$1000  <$2000  <$3000  <$4000  >$4000  1  2  3  4  >=5  0  1  2  3  >=4  None  Private  Public  Medicare  Yes  No  Yes  No  SNAP/food stamps  WIC  Other  None  Yes  No  Urban  Rural  Suburban  Current  Former  Never  0  Daily  4-6d/w  2-3d/w  1d/w  Yes  No  Yes  No  0  Daily  4-6d/w  2-3d/w  1d/w | 81  38  63  106  96  40  19  92  41  25  53  56  45  100  55  131  44  31  27  209  34  26  14  5  4  143  39  101  26  260  69  217  32  0  5  249  28  258  55  102  124  33  107  148  251  30  3  2  1  10  278  117  171  172  19  16  29  50 | 28.1  13.2  21.9  36.8  33.3  13.9  6.6  31.9  14.2  9.0  19.0  20.1  16.1  35.8  19.1  45.5  15.3  10.8  9.4  72.6  11.8  9.0  4.9  1.7  1.4  49.8  13.6  35.2  9.1  90.9  24.1  75.9  11.2  0.0  1.7  87.1  9.8  90.2  19.6  36.3  44.1  11.5  37.2  51.4  87.5  10.5  1.0  0.7  0.3  3.5  96.5  40.6  59.4  60.1  6.6  5.6  10.1  17.5 |

| **Disease Characteristics** | **Categories** | **n** | **%** |
| --- | --- | --- | --- |

| *Type of cancer diagnose with*  *Time since disease diagnosis*  *Stage of disease*  *Type of cancer treatment received?* | Breast  Prostate  Ovarian  Cervical  Colon/Rectum  Lung  Other  0-6 months  7 months-less than 2 years  2 years-less than 5 years  5 years or more  I  II  III  IV  Unknown  Chemotherapy  Radiation therapy  Hormone therapy  Surgery  Combination therapy  Other | 105  43  14  15  31  11  53  184  43  41  19  24  56  47  84  72  86  15  0  2  184  1 | 38.6  15.8  5.1  5.5  11.4  4.0  19.5  64.1  15.0  14.3  6.6  8.5  19.8  16.6  29.7  25.4  29.9  5.2  0.0  0.7  63.9  0.3 |
| --- | --- | --- | --- |

| **Medication and Treatment Adherence** | **Categories** | **n** | | **%** | |  |
| --- | --- | --- | --- | --- | --- | --- |
|  |  |  | |  | |  |
| *Do you receive oral medications as part of your cancer treatment?*  *During the past 4 days, on how many days have you missed taking all your doses?*  *How closely did you follow your specific medication schedule over the last four days?*  *Do any of your medications have special instructions?*  *If yes, how often did you follow those special instructions over the last four days?*  *Did you miss any of your cancer medications last weekend—last Saturday or Sunday?*  *When was the last time you missed any of your medications?*  *Were away from home*  *Were busy with other things?*  *Simply forgot?*  *Had too many pills to take?*  *Wanted to avoid side effects?*  *Didn’t want others to notice you taking meds?*  *Had a change in daily routine?*  *Felt like the drug was toxic/harmful?*  *Fell asleep/slept through dose time?*  *Felt sick or ill?*  *Felt depressed/overwhelmed?*  *Had problems taking pills at specified times?*  *Ran out of pills?*  *Felt good?*  *Have you ever missed your treatment appointments?*  *If yes, how many times within the past treatment cycle?* | Yes  No  None  One day  Two days  Three days  Four days  Never  Some of the time  About half of the time  Most of the time  All of the time  Yes  No  Never  Some of the time  About half of the time  Most of the time  All of the time  Yes  No  Within the past week  1-2 weeks ago  2-4 weeks ago  1-3 months ago  More than 3 months ago  Never skip or NA  Never  Rarely  Sometimes  Often  Never  Rarely  Sometimes  Often  Never  Rarely  Sometimes  Often  Never  Rarely  Sometimes  Often  Never  Rarely  Sometimes  Often  Never  Rarely  Sometimes  Often  Never  Rarely  Sometimes  Often  Never  Rarely  Sometimes  Often  Never  Rarely  Sometimes  Often  Never  Rarely  Sometimes  Often  Never  Rarely  Sometimes  Often  Never  Rarely  Sometimes  Often  Never  Rarely  Sometimes  Often  Never  Rarely  Sometimes  Often  Yes  No  0-1  2-4  >5 | | 131  156  142  11  0  0  1  9  1  2  40  97  96  54  3  5  1  23  73  5  148  17  12  11  12  14  75  109  25  17  1  104  32  14  0  87  41  18  3  131  8  8  3  119  17  6  6  141  7  1  0  108  26  14  0  126  15  6  2  110  22  13  3  107  26  12  4  126  16  6  1  123  16  6  3  116  20  12  1  122  13  10  4  15  271  284  3  1 | | 45.6  54.4  92.2  7.1  0.0  0.0  0.6  6.0  0.7  1.3  26.8  65.1  64.0  36.0  2.9  4.8  1.0  21.9  69.5  3.3  96.7  12.1  8.5  7.8  8.5  9.9  53.2  71.7  16.4  11.2  0.7  69.3  21.3  9.3  0.0  58.4  27.5  12.1  2.0  87.3  5.3  5.3  2.0  80.4  11.5  4.1  4.1  94.6  4.7  0.7  0.0  73.0  17.6  9.5  0.0  84.6  10.1  4.0  1.3  74.3  14.9  8.8  2.0  71.8  17.4  8.1  2.7  84.6  10.7  4.0  0.7  83.1  10.8  4.1  2.0  77.9  13.4  8.1  0.7  81.9  8.7  6.7  2.7  5.2  94.8  98.6  1.0  0.3 | |

| **Quality of Life** | **Categories** | **n** | **%** |
| --- | --- | --- | --- |
|  |  |  |  |
| *During the past week, did you have dry mouth?*  *During the past week, did food and drink taste different than usual?*  *During the past week, were your eyes painful, irritate, or watery?*  *During the past week, have you lost any hair?*  *During the past week, were you upset by the loss of your hair?*  *During the past week, did you feel ill or unwell?*  *During the past week, did you have hot flashes?*  *During the past week, did you have headaches?*  *During the past week, have you felt physically less attractive as a result of your disease or treatment?*  *During the past week, have you been feeling less feminine as a result of your disease or treatment?*  *During the past week, did you find it difficult to look at yourself naked?*  *During the past week, have you been dissatisfied with your body?*  *During the past week, were you worried about your health in the future?*  *During the past four weeks, to what extent were you interested in sex?*  *During the past four weeks, to what extent were you sexually active (with or without intercourse)?*  *During the past four weeks, to what extent was sex enjoyable for you?*  *During the past week, did you have any pain in your arm or shoulder?*  *During the past week, did you have a swollen arm or hand?*  *During the past week, was it difficult to raise your arm or to move it sideways?*  *During the past week, have you had any pain in the area of your breast?*  *During the past week, was the area of your affected breast swollen?*  *During the past week, was the area of your affected breast oversensitive?*  *During the past week, have you had skin problems on or in the area of your affected breast (e.g. itchy, dry, flaky)?* | Not at all  A little  Quite a bit  Very much  Not at all  A little  Quite a bit  Very much  Not at all  A little  Quite a bit  Very much  Not at all  A little  Quite a bit  Very much  Not at all  A little  Quite a bit  Very much  Not at all  A little  Quite a bit  Very much  Not at all  A little  Quite a bit  Very much  Not at all  A little  Quite a bit  Very much  Not at all  A little  Quite a bit  Very much  Not at all  A little  Quite a bit  Very much  Not at all  A little  Quite a bit  Very much  Not at all  A little  Quite a bit  Very much  Not at all  A little  Quite a bit  Very much  Not at all  A little  Quite a bit  Very much  Not at all  A little  Quite a bit  Very much  Not at all  A little  Quite a bit  Very much  Not at all  A little  Quite a bit  Very much  Not at all  A little  Quite a bit  Very much  Not at all  A little  Quite a bit  Very much  Not at all  A little  Quite a bit  Very much  Not at all  A little  Quite a bit  Very much  Not at all  A little  Quite a bit  Very much  Not at all  A little  Quite a bit  Very much | 100  113  44  30  98  96  43  49  165  81  26  13  117  55  24  88  74  52  26  35  120  120  31  13  169  63  33  18  130  114  30  11  116  95  40  33  122  57  23  18  172  75  14  20  124  100  31  28  38  104  89  47  129  101  33  15  153  84  28  10  21  42  42  31  188  63  21  10  249  24  8  1  226  34  12  8  75  47  4  5  102  18  8  3  97  23  9  3  108  15  8  3 | 34.8  39.4  15.3  10.5  34.4  33.6  15.0  17.1  57.9  28.4  9.1  4.6  41.2  19.4  8.5  31.0  39.6  27.8  13.9  18.7  42.3  42.3  10.9  4.6  59.7  22.3  11.7  6.4  45.6  40.0  10.5  3.9  40.8  33.5  14.1  11.6  55.5  25.9  10.5  8.2  61.2  26.7  5.0  7.1  43.8  35.3  11.0  9.9  13.7  37.4  32.0  16.9  46.4  36.3  11.9  5.4  55.6  30.5  10.2  3.6  15.4  30.9  30.9  22.8  66.7  22.3  7.4  3.5  88.3  8.5  2.8  0.4  80.7  12.1  4.3  2.9  57.3  35.9  3.1  3.8  77.9  13.7  6.1  2.3  73.5  17.4  6.8  2.3  80.6  11.2  6.0  2.2 |

| **Risk Perception, Attitudes, and Behaviors** | **Categories** | **n** | **%** |
| --- | --- | --- | --- |
|  |  |  |  |
| ***Risk Perception***  *I think contamination of food by bacteria or viruses is a serious problem*  *I am knowledgeable about how to keep the food I prepare and eat at home safe*  *I am knowledgeable about safe food choices when I eat outside the home*  *Because I am a cancer survivor I am at an increased risk of getting foodborne illness or food poisoning*  ***Attitudes***  *I am not concerned if I thaw perishable foods on the kitchen counter*  *Cooking and eating eggs that have firm yolks and whites is important to me for safety*  *Drinking pasteurized apple juice or cider is important to me for safety*  *After cutting raw meat or chicken, I like to wash the cutting board, knife, and counter top with hot soapy water before continuing cooking*  *I am not interested in using a meat thermometer*  *I don’t worry that I might get sick if I eat alfalfa and other raw sprouts*  *I am worried that I may get sick if I eat hot dogs right out of the package*  *Using cheese and yogurt made only from pasteurized milk is important to me*  *I am concerned that I may get sick if I eat raw oysters*  *I don’t worry about keeping my refrigerator at or below 40 degrees Fahrenheit*  *I don’t worry about washing my hands after playing with my pets*  *It is not important cover a cut or sore on my hand before I prepare food*  *Refrigerating food such as rice and beans overnight before serving them the following day is not important to me*  *There is no need to store eggs in a refrigerator, room temperature is fine*  ***Behaviors***  *I wash my hands with soap and warm running water before preparing food*  *After playing with a pet, and before getting a snack, I wash my hands with soap and warm running water*  *After cutting raw meat, chicken, or seafood, I wash all items that came into contact with the raw food (e.g. cutting board, knife, counter top) with hot, soapy water before I continue cooking*  *I thoroughly rinse fresh vegetables under running water before eating them*  *I wash the plate used to hold raw meat, poultry, or seafood with hot, soapy water before returning cooked food to the plate OR I use a clean plate*  *I wash my hands with soap and warm running water after working with raw meat, chicken, or seafood and before I continue cooking*  *I clean countertops with hot, soapy water after preparing food*  *I leave cooked foods, such as rice or beans, on the stovetop overnight to be used the next day*  *I put frozen meat and poultry on the counter in the morning, so it will be thawed and ready to cook in the evening*  *I store my eggs at room temperature*  *When I cook fish, I check that the flesh flakes easily with a fork before serving*  *I use a thermometer to check the temperature of my fridge*  *I prepare food for others when I have diarrhea*  *I use a thermometer to determine if leftovers have been reheated enough*  *I use a thermometer to determine if chicken breasts have been cooked enough* | Strongly Disagree  Disagree  Neutral  Agree  Strongly Agree  Strongly Disagree  Disagree  Neutral  Agree  Strongly Agree  Strongly Disagree  Disagree  Neutral  Agree  Strongly Agree  Strongly Disagree  Disagree  Neutral  Agree  Strongly Agree  Never  Rarely  Some of the Time  Most of the Time  Always  Never  Rarely  Some of the Time  Most of the Time  Always  Never  Rarely  Some of the Time  Most of the Time  Always  Never  Rarely  Some of the Time  Most of the Time  Always  Never  Rarely  Some of the Time  Most of the Time  Always  Never  Rarely  Some of the Time  Most of the Time  Always  Never  Rarely  Some of the Time  Most of the Time  Always  Never  Rarely  Some of the Time  Most of the Time  Always  Never  Rarely  Some of the Time  Most of the Time  Always  Never  Rarely  Some of the Time  Most of the Time  Always  Never  Rarely  Some of the Time  Most of the Time  Always  Never  Rarely  Some of the Time  Most of the Time  Always  Never  Rarely  Some of the Time  Most of the Time  Always  Never  Rarely  Some of the Time  Most of the Time  Always  Never  Rarely  Some of the Time  Most of the Time  Always  Never  Rarely  Some of the Time  Most of the Time  Always  Never  Rarely  Some of the Time  Most of the Time  Always  Never  Rarely  Some of the Time  Most of the Time  Always  Never  Rarely  Some of the Time  Most of the Time  Always  Never  Rarely  Some of the Time  Most of the Time  Always  Never  Rarely  Some of the Time  Most of the Time  Always  Never  Rarely  Some of the Time  Most of the Time  Always  Never  Rarely  Some of the Time  Most of the Time  Always  Never  Rarely  Some of the Time  Most of the Time  Always  Never  Rarely  Some of the Time  Most of the Time  Always  Never  Rarely  Some of the Time  Most of the Time  Always  Never  Rarely  Some of the Time  Most of the Time  Always  Never  Rarely  Some of the Time  Most of the Time  Always  Never  Rarely  Some of the Time  Most of the Time  Always | 18  16  52  99  103  9  2  14  116  147  9  5  33  134  107  15  23  101  80  62  76  64  52  37  58  53  41  54  55  83  82  48  30  45  77  9  12  15  49  200  71  64  63  43  46  104  52  43  25  54  86  48  27  26  97  60  42  35  53  94  88  23  11  28  126  107  34  30  34  80  120  26  22  22  92  109  17  18  16  126  122  32  21  19  88  203  14  11  12  44  4  1  9  31  238  9  8  19  43  200  9  7  7  27  233  6  1  21  48  209  4  1  3  15  261  5  1  6  23  249  5  3  18  54  205  240  15  11  3  15  128  43  56  23  34  244  15  7  2  16  41  23  30  39  148  119  37  34  22  71  165  60  33  7  16  171  43  28  17  24  125  32  29  33  64 | 6.3  5.6  18.1  34.4  35.8  3.1  0.7  4.9  40.3  51.0  3.1  1.7  11.5  46.5  37.2  5.3  8.2  35.9  28.5  22.1  26.5  22.3  18.1  12.9  20.2  18.5  14.3  18.9  19.2  29.0  29.1  17.0  10.6  16.0  27.3  3.2  4.2  5.3  17.2  70.2  24.7  22.3  22.0  15.0  16.0  37.4  18.7  15.5  9.0  19.4  30.3  16.9  9.5  9.2  34.2  21.1  14.8  12.3  18.7  33.1  31.9  8.3  4.0  10.1  45.7  37.5  11.9  10.5  11.9  28.1  42.6  9.2  7.8  7.8  32.6  38.1  5.9  6.3  5.6  44.1  43.3  11.3  7.4  6.7  31.2  71.5  4.9  3.9  4.2  15.5  1.4  0.4  3.2  11.0  84.1  3.2  2.9  6.8  15.4  71.7  3.2  2.5  2.5  9.5  82.3  2.1  0.4  7.4  16.8  73.4  1.4  0.4  1.1  5.3  91.9  1.8  0.4  2.1  8.1  87.7  1.8  1.1  6.3  18.9  71.9  84.5  5.3  3.9  1.1  5.3  45.1  15.1  19.7  8.1  12.0  85.9  5.3  2.5  0.7  5.6  14.6  8.2  10.7  13.9  52.7  42.0  13.1  12.0  7.8  25.1  58.7  21.4  11.7  2.5  5.7  60.4  15.2  9.9  6.0  8.5  44.2  11.3  10.2  11.7  22.6 |
|  |  |  |  |

| **Food Preferences** | **Categories** | **n** | **%** |
| --- | --- | --- | --- |
|  |  |  |  |
| *Do you eat rare hamburger*  *Do you eat eggs with runny yolks*  *Do you eat raw oysters/oysters on the half shell*  *Do you eat raw fish*  *Do you eat homemade cookie dough?*  *Do you eat alfalfa or other raw sprouts*  *Do you eat ceviche (marinated raw fish)*  *Do you eat sushi (made with raw fish)*  *Do you eat at restaurant salad bars*  *Do you eat cold hot dogs*  *Do you eat soft cheese like Brie, Camembert, and queso fresco*  *Do you eat smoked fish served cold without reheating*  *Do you eat cold deli meats* | Yes  No  Yes  No  Yes  No  Yes  No  Yes  No  Yes  No  Yes  No  Yes  No  Yes  No  Yes  No  Yes  No  Yes  No  Yes  No | 25  261  159  127  37  249  28  258  93  193  64  222  22  264  46  238  197  88  43  240  110  175  39  246  252  33 | 8.7  91.3  55.6  44.4  12.9  87.1  9.8  90.2  32.5  67.5  22.4  77.6  7.7  92.3  16.2  83.8  69.1  30.9  15.2  84.8  38.6  61.4  13.7  86.3  88.4  11.6 |

| **Food Safety Knowledge** | **Categories** | **n** | **%** |
| --- | --- | --- | --- |
|  |  |  |  |
| ***General Food Safety***  *All harmful bacteria are destroyed by thorough and complete cooking*  *Moldy hard cheddar cheese is safe to eat if you scratch the mold off the surface of the cheese*  *Organically grown produce is less likely to cause foodborne illness than conventionally grown produce*  *Pesticide residues are the most serious food safety problem*  *Young children are more vulnerable to foodborne illnesses than teenagers or adolescents*  *Unsafe foods can be identified by the way they look and smell*  *Food allergies are a serious food safety problem*  *Foodborne illness outbreaks are associated with eating all types of food*  *Bacteria and viruses found in food can make you sick*  *Disease-causing bacteria can be found on food*  *It can take only a small number of harmful bacteria to make a person sick*  ***Cross-Contamination (Separation)***  *After cutting up raw meat or chicken, you should wipe off the cutting board with a wet dishcloth or sponge before using the board to cut produce*  *It is safe to store fresh produce below raw meat and poultry in the fridge*  *It is important to wash hands after cracking an egg*  *When grocery shopping, raw meat, fish, and/or poultry should be packed separately from ready-to-eat foods from the deli or produce area*  *In the kitchen, food can become contaminated with harmful bacteria during handling and storage*  *The sauce that was used to marinate raw chicken can be refrigerated and used again safely*  *Since a food thermometer has a metal stem, it does not need to be sanitized after using it*  *It is safe to place cooked meat on the same unwashed plate you used for uncooked meat*  ***Food Preparation***  *To be safe to eat, the temperature of stuffing cooked inside a turkey should be at least 145 degrees Fahrenheit*  *Chicken breasts should be cooked until the temperature in the middle is 165 degrees Fahrenheit*  *Cooked rice held at room temperature for more than 4 hours is safe to eat*  *I can always tell that my hamburger is completely cooked by its color*  *It is safe to use unpasteurized eggs in recipes that will not be cooked*  *Cooked meat held at room temperature for more than 2 hours is safe to eat*  *Using a food thermometer is the best way of knowing that food is thoroughly cooked*  *It is safe to eat raw cookie dough or cake batter that contains raw eggs*  ***Food Storage (Chill)***  *One of the most common causes of foodborne illness is failure to properly cool food*  *Deli meats or cold cuts sliced at the deli counter are safe to eat for seven days after purchase*  *It is safe to leave hot, thoroughly cooked food on the counter to completely cool to room temperature before putting it in the refrigerator*  *Meat that has been handled and/or prepared properly can be kept in the freezer for 6 months and still be safe to eat*  *The temperature of a home refrigerator should be at 40 degrees Fahrenheit or below*  *Disease-causing bacteria can survive and/or grow at refrigerator temperatures*  *It is safe to leave meat on the counter to thaw*  *If a green bean casserole is left on the kitchen counter overnight, it is safe to eat if it is properly reheated*  ***Clean Up (Cleaning/Hygiene)***  *Countertops may be sanitized by washing with soap and water*  *Hand sanitizers are the best way to wash your hands*  *You should wash the outside of a cantaloupe before cutting it*  *You should wash your hands with warm, soapy water for at least 20 seconds before starting to prepare food*  *It is safe to eat a snack while you are preparing food*  *If you use a dishcloth to wipe up liquid from raw meat or chicken, it is safe to use the cloth for washing dishes if you rinse the cloth in hot water*  *Sponges will not be contaminated with bacteria since they are used to wash utensils with soap and water*  *It is safe to use the same spoon to taste and then stir the food without washing the spoon*  *It is safe to use a cloth towel to clean up spills on kitchen surfaces and then use it to dry off washed fresh fruits or vegetables*  *After handling raw meat, fish, and/or poultry, wiping hands on a paper towel is sufficient to clean hands* | Agree  Disagree  Agree  Disagree  Agree  Disagree  Agree  Disagree  Agree  Disagree  Agree  Disagree  Agree  Disagree  Agree  Disagree  Agree  Disagree  Agree  Disagree  Agree  Disagree  Agree  Disagree  Agree  Disagree  Agree  Disagree  Agree  Disagree  Agree  Disagree  Agree  Disagree  Agree  Disagree  Agree  Disagree  Agree  Disagree  Agree  Disagree  Agree  Disagree  Agree  Disagree  Agree  Disagree  Agree  Disagree  Agree  Disagree  Agree  Disagree  Agree  Disagree  Agree  Disagree  Agree  Disagree  Agree  Disagree  Agree  Disagree  Agree  Disagree  Agree  Disagree  Agree  Disagree  Agree  Disagree  Agree  Disagree  Agree  Disagree  Agree  Disagree  Agree  Disagree  Agree  Disagree  Agree  Disagree  Agree  Disagree  Agree  Disagree  Agree  Disagree | 209  75  100  183  98  183  187  95  189  93  157  127  248  34  193  87  276  9  264  17  274  10  170  113  53  229  214  69  271  13  277  8  20  265  17  266  7  278  177  97  248  27  80  202  152  132  41  237  79  202  261  23  44  239  181  100  171  113  189  93  215  68  263  18  222  57  84  197  21  260  226  57  64  218  215  66  265  18  112  169  31  250  22  258  16  265  13  268  10  271 | 73.6  26.4  35.3  64.7  .  34.9  65.1  66.3  33.7  67.0  33.0  55.3  44.7  87.9  12.1  68.9  31.1  96.8  3.2  94.0  6.0  96.5  3.5  60.1  39.9  18.8  81.2  75.6  24.4  95.4  4.6  97.2  2.8  7.0  93.0  6.0  94.0  2.5  97.5  64.6  35.4  90.2  9.8  28.4  71.6  53.5  46.5  14.7  85.3  28.1  71.9  91.9  8.1  15.5  84.5  64.4  35.6  60.2  39.8  67.0  33.0  76.0  24.0  93.6  6.4  79.6  20.4  29.9  70.1  7.5  92.5  79.9  20.1  22.7  77.3  76.5  23.5  93.6  6.4  39.9  60.1  11.0  89.0  7.9  92.1  5.7  94.3  4.6  95.4  3.6  96.4 |

| **Food Acquisition Practices** | **Categories** | **n** | **%** |
| --- | --- | --- | --- |
|  |  |  |  |
| *I cook with other people*  *I borrow food from other people*  *I get food from my workplace*  *I acquire discarded food*  *I acquire food from private individuals*  *I seek roadkill*  *I hunt or fish*  *I purchase food from private individuals*  *I purchase expired foods*  *I purchase nearly expired foods*  *I purchase foods in dented or damaged packages*  *I remove slime from lunch meat*  *I remove mold from cheese*  *I remove mold from grains*  *I remove insects from grains*  *I remove spoiled pars of fruits/vegetables*  *I store perishables inadequately*  *I eat spoiled food*  *I eat expired food*  *I eat non-food items*  *I eat other people’s leftovers*  *I eat roadkill*  *I eat pet food* | Never  Rarely  Some of the time  Most of the time  Always  Never  Rarely  Some of the time  Most of the time  Always  Never  Rarely  Some of the time  Most of the time  Always  Never  Rarely  Some of the time  Most of the time  Always  Never  Rarely  Some of the time  Most of the time  Always  Never  Rarely  Some of the time  Most of the time  Always  Never  Rarely  Some of the time  Most of the time  Always  Never  Rarely  Some of the time  Most of the time  Always  Never  Rarely  Some of the time  Most of the time  Always  Never  Rarely  Some of the time  Most of the time  Always  Never  Rarely  Some of the time  Most of the time  Always  Never  Rarely  Some of the time  Most of the time  Always  Never  Rarely  Some of the time  Most of the time  Always  Never  Rarely  Some of the time  Most of the time  Always  Never  Rarely  Some of the time  Most of the time  Always  Never  Rarely  Some of the time  Most of the time  Always  Never  Rarely  Some of the time  Most of the time  Always  Never  Rarely  Some of the time  Most of the time  Always  Never  Rarely  Some of the time  Most of the time  Always  Never  Rarely  Some of the time  Most of the time  Always  Never  Rarely  Some of the time  Most of the time  Always  Never  Rarely  Some of the time  Most of the time  Always  Never  Rarely  Some of the time  Most of the time  Always | 43  83  132  20  7  160  91  31  1  2  191  43  38  9  3  258  17  5  3  1  163  56  61  1  2  278  3  1  1  1  221  29  24  7  3  207  49  26  1  2  256  22  6  0  1  169  75  39  0  0  196  53  33  1  1  246  9  3  8  17  181  46  31  6  20  253  8  2  0  20  239  6  6  1  31  153  31  51  6  43  223  38  11  3  9  274  9  1  0  1  223  45  13  2  1  268  8  5  0  3  194  63  24  2  2  282  1  1  0  1  282  2  0  0  1 | 15.1  29.1  46.3  7.0  2.5  56.1  31.9  10.9  0.4  0.7  67.3  15.1  13.4  3.2  1.1  90.8  6.0  1.8  1.1  0.4  57.6  19.8  21.6  0.4  0.7  97.9  1.1  0.4  0.4  0.4  77.8  10.2  8.5  2.5  1.1  72.6  17.2  9.1  0.4  0.7  89.8  7.7  2.1  0.0  0.4  59.7  26.5  13.8  0.0  0.0  69.0  18.7  11.6  0.4  0.4  86.9  3.2  1.1  2.8  6.0  63.7  16.2  10.9  2.1  7.0  89.4  2.8  0.7  0.0  7.1  84.5  2.1  2.1  0.4  11.0  53.7  11.0  18.0  2.1  15.2  78.5  13.4  3.9  1.1  3.2  96.1  3.2  0.4  0.0  0.4  78.5  15.8  4.6  0.7  0.4  94.4  2.8  1.8  0.0  1.1  68.1  22.1  8.4  0.7  0.7  98.9  0.4  0.4  0.0  0.4  98.9  0.7  0.0  0.0  0.4 |
